# Stunting, Beyond Acute Diarrhoea: *Giardia Duodenalis*, in Cambodia

**DOI:** 10.3390/nu10101420

**Published:** 2018-10-03

**Authors:** Yannick Caron, Rathmony Hong, Ludovic Gauthier, Arnaud Laillou, Frank T. Wieringa, Jacques Berger, Etienne Poirot

**Affiliations:** 1 Institut Pasteur du Cambodge, Laboratory of Medical Biology, 5 Boulevard Monivong, P.O. Box 983, Phnom Penh 12100, Cambodia; ycaron@pasteur-kh.org; 2 United Nations Children’s Fund (UNICEF), Integrated Early Childhood Development, Exchange Square, 5th Floor, No. 19&20, Street 106, Sangkat Wat Phnom, Khan Daun Penh, Phnom Penh 12100, Cambodia; rhong@unicef.org (R.H.); alaillou@unicef.org (A.L.); 3 Independent consultant, Phnom Penh 12100, Cambodia; gauthier.ludo@hotmail.fr; 4 Institute of Research for Development (IRD), UMR Nutripass IRD-UM2-UM1, 3400 Montpellier, France; franck.wieringa@ird.fr (F.T.W.); jacques.berger@ird.fr (J.B.)

**Keywords:** *Giardia duodenalis*, diarrhea, livestock ownership, feces, stunting

## Abstract

Background: The adverse outcomes of malnutrition on the development of a child are well acknowledged as are the broad variety of contextual factors that may impact child nutritional status. Adequate nutrient intake and the adoption of appropriate water, sanitation and hygiene measures are largely documented for their positive influence on health. Improved sanitation and protection from human feces can significantly lower the incidence of diarrhea and environmental enteropathy. However, the impact of excessive exposure to animal feces on child health is less well documented. Objectives: This study tests the hypothesis that there is a positive association between exposure to animal feces, morbidity and anthropometric outcomes in children under 5 years of age, in Cambodia. It aims to improve insights that can contribute to discerning high-impact policies that promote children can develop to their full potential. Methods: Data for this study was drawn from the third follow-up round of the MyHealth project cohort study that is conducted in six districts of three Cambodian provinces (Phnom Penh, Kratie and Ratanak Kiri). The analysis included a sample of 639 children under 5 years of age. Results: The presence of livestock and more particularly, pigs near the main household dwelling was found a risk factor associated with *Giardia duodenalis* infection (23%). *Giardia duodenalis* infection was found to be a protective factor for acute diarrhea, yet, associated with stunting in the univariate model. Conclusions: Preventive measures that protect from extensive exposure to animal feces may be most effective to prevent infection with *Giardia duodenalis* and consequent stunting, thereby improving the potential for a healthy development in young Cambodian children. The results support the need for cross-sector policy measures that reinforce comprehensive early childhood interventions towards improving nutritional status as part of a wider set of child welfare and development measures.

## 1. Background

Over the last decades, Cambodia has made significant improvements in key health indicators. Yet, stunting in children remains high (32.4% of children below the age of five years, in 2014) and continues to considerably affect their health and development potential [1,2]. Interventions to tackle stunting in children mainly focus on evidence that promotes adequate nutrient intake and the adoption of appropriate water-sanitation-hygiene (WASH) measures to prevent contamination with pathogens from human feces that have been strongly associated with diarrheal illnesses in children [3].

The impact of exposure to animal feces on nutrition status has been little explored [4,5] even exposure to animal feces may be common in low-income countries (LIC) where animals, both domestic and livestock frequently share the household dwelling. Recent literature highlights the importance of zoonotic pathogens on human health. It is estimated that nearly two-thirds of human pathogens (61% of 1415 pathogens known to infect humans) and three quarters of emerging pathogens are of zoonotic origin [6]. Exposure to poultry has been positively associated with an increased risk of respiratory infections in both, adults and young children [7] and a recent meta-analysis that re-examined linkages between exposure to animals and diarrhoea in young children found that 21 out of 27 suitable studies reported significant positive associations [8].

Among the pathogens involved in digestive disorders, certain parasite infections remain a serious threat. Though, studies that explore intestinal protozoal and soil-transmitted helminth infection prevalence in impoverished regions of the world remain limited. *Giardia duodenalis* (syn. *Giardia intestinalis*, *Giardia lamblia*; abbr. *G. duodenalis*) is found the most common intestinal parasite in humans in developed countries and has a global distribution. In Asia, Africa and Latin America, about 200 million people were identified with symptomatic *Giardiasis* with about 500,000 new cases reported each year [9]. The presence of *Giardia duodenalis* has also been noted in domestic animals, and more particularly in livestock, domestic dogs and cats and numerous species of wild mammals [10].

Exposure to animal feces was identified as a potential risk factor for intestinal infections in children and their caregivers in a study by Zambrano [8]. In this study modest evidence related exposure to livestock and animal feces with an increased probability of diarrheal infections. Furthermore, it has been hypothesized that exposure to animal feces may increase high concentrations of bacteria—even non-pathogenic bacteria—in the small intestines and be responsible for chronic subclinical damage and environmental enteric dysfunction (EED) leading to stunting [11,12,13].

This study explores associations between exposure of young children to animal feces and nutritional status with the aim to improve insights that may contribute to discerning policies that promote children to develop to their full potential. We considered exposure to animal feces and nutritional status against a series of co-variables regarding morbidity and socio-economic characteristics. We have chosen Cambodia as setting, because of its high rate of stunting in children under 5 years of age (prevalence 32.4%) [14], limited adoption of good hygiene and nutrition practices and routine presence of livestock at residence allowing for the identification of relevant associations [15,16].

## 2. Methods

### 2.1. Study Design and Sampling

Data for this study was drawn from the third follow-up round of the MyHealth project cohort study. The main objective of this project is to collect in-depth data for at least 3 years on health and nutritional status to better inform the government on progresses that can be made with enhanced health monitoring. At baseline, a sample size of 1200 children under 3 years of age per site was calculated required to observe a reduction in child stunting from 32% to 26% over a 3 to 5 years period (with a precision of 3% and a dropout of 20%). Local midwives and village health volunteers provided a list with the names of all children under 3 years of age in the selected areas. This list served as a sampling frame for the sample selection process. Households from children under the age of 3 years were randomly designated to the project using random tables. Data from two selected provinces (Kratie and Ratanak Kiri) were included in this study, data from the capital Phnom Penh was not included due to the low prevalence of domestic and livestock animals at household level.

A sample size of 682 children was calculated required to get a precision of 3% with a type 1 error of 5% using a prevalence of *Giardiasis* of 20% based on a previous study on *G. duodenalis* infection in a rural village, in Cambodia [17]. After data collection and cleaning, a final sample size of 639 subjects was available for analysis, allowing to estimate *G. duodenalis* infection prevalence with a precision of 3.1%. Data was collected through interviews with the mothers of the selected children and a series of anthropometric measures. Some mothers attended the interview but did not attend anthropometric data collection. Information on anthropometric status was available for 593 children in total (Kratie *n* = 302; Ratanak Kiri *n* = 291). This latter sample was used for the analysis of malnutrition indicators.

### 2.2. Study Indicators 

Gender, weight, height, and Mid-upper Arm Circumference (MUAC) were recorded for all children. Height was measured by field workers using UNICEF height boards with standing plates and moveable head boards and accuracy to 1 mm. Weight was measured using calibrated digital balances (SECA, Hamburg, Germany) with 100 g precision. MUAC of children was measured using a plastic, colored, insertion tape (marked in millimeters, with cut-off points from red to yellow at 110 mm and from yellow to green at 125 mm; incapable of stretching and unresponsive to temperatures; supplied by UNICEF Copenhagen). We calculated height-for-age Z-scores (HAZ) and weight-for-age Z-scores (WAZ) using WHO 2006 standards for children 0–59 months [17] and WHO Anthro software (version 3.2.2, January 2011, World Health Organization, 1211 Geneva 27, Switzerland). Z-scores < −2 SD for length/height-for-age was defined as stunting while wasting was weight-for-length/height < −2 SD and MUAC below 115 mm [18]. 

One day before the interview, flasks were given to the mothers included in the study sample in order to collect a stool sample of participating children. The flasks were returned to the interviewers the next day, preserved in a 10% formalin solution and sent for analysis to the Pasteur Institute in Phnom Penh. Stool samples were examined using a coproscopic technique. Simple qualitative direct examination analysis was conducted to highlight the presence of cysts of *G. duodenalis* (microscopic examination). *Giardia* infection was categorized as presence/absence of *G. duodenalis* cysts. Acute diarrhea episodes were measured through maternal recall (three or more loose stools passed in a 24-h period) with at least one episode in the two weeks before the interview.

### 2.3. Covariates

The main covariates for this study were: type of livestock ownership, sanitation facilities, drinking water source and place where the child usually defecated. Information on these indicators were asked directly to the mother during the interview. The different types of livestock included pig, cow and chicken. Sanitation facilities were categorized into improved and non-improved as per WHO definitions. Improved sanitation facilities included: flush toilets, ventilated improved pit latrines and pit latrines with slabs. Source of drinking water was defined as improved if the household used: piped water, public tap or standpipe, tube well or borehole, protected dug well or spring (public or private), or bottled water in line with the categorization used in the Cambodia Demographic Health Survey (CDHS). Place where the child usually defecated, included: yard, own clothes, diaper, latrine and potty. Yard and own clothes were considered as unsafe defecation places. Child factors, mother education and household wealth index were considered as potential cofounders. Child factors included age in months and gender. Mother education was categorized into three categories: no education/informal schooling, primary education and secondary or more. Household socio-economic status was represented through a wealth index. This index was calculated using the baseline data of the survey. Its calculation was done through principal component analysis with variables on ownership of house and land, housing quality and household assets, as described by Filmer and Pritchett [18] and the first principal component to be divided into quintiles. For this study, the two first quintiles were grouped together as well as the two last ones, leading to an index with three categories: poor, middle class and rich. Finally, as models were fitted on the full sample (grouping the two provinces together) a region indicator variable was systematically included in the models.

### 2.4. Statistical Analysis

Data management and statistical analysis were done using STATA version 13 (Stata Corp., College Station, TX, USA) and R software version 3.4.0 (Free Software Foundation (FSF), MA, USA). First, descriptive analyses were conducted to examine different background characteristics and distribution factors across the two regions. Associations were examined between *G. duodenalis* infection and ownership of different types of livestock, WASH factors, age, mother education and wealth index using a multivariate logit model. All selected variables were included in the final model. A focus was made on the association between *G. duodenalis* infection and three other outcomes (symptoms of diarrhea, HAZ and WHZ), including them as responses and the presence of *G. duodenalis* as the main independent variable. The binary diarrhea variable was analyzed using a multivariate logit model whereas linear regression models were fitted for the two continuous anthropometric variables. For these three models, all the covariates were considered at the first stage of model building. To build final models parsimonious with control for cofounding effect, the selection among these variables was done using the method as proposed by Hosmer and Lemeshow [19].

In addition to controlling all our models for region and age of the child; the two anthropometric models also had gender, age and age squared included as forced variables. To facilitate comparisons across variables included in all regression models, both, unadjusted (bivariate) and adjusted (multivariate) coefficients were shown. When the distribution of both, the outcome and the included covariate differed considerably between regions (Kratie and Ratanak Kiri), a univariate analysis for this covariate was done adjusting for region. For all analyses, the type I error risk was 0.05.

### 2.5. Ethical Approval

Ethical approval for the study was obtained from the Cambodia National Ethical Committee for Health Research (117/NECHR). Informed consent was obtained from all participants, with consent obtained from parents or guardians for participating children.

## 3. Results

### 3.1. Descriptive Analysis

Table 1, provides an overview of selected sample characteristics. Combining the two regions together (*N* = 639), a quarter of the children (23.0%) presented *G. duodenalis* infection. The mean child’s age was 20.6 months, gender distribution was 49.6% boys. Wealth index identified 50.2% of households as poor and 22% as rich. Households owned pigs, cows and chicken at a rate of 39.1%, 39.9% and 14% respectively.

A majority of mothers had primary education only (45.5%) with education of the remaining mothers almost equally distributed across no education and secondary education or more (27.7% and 26.8% respectively). One-third of households reported improved sanitation facilities and children defecating in a safe place. Drinking water was reported to come from an improved source by 56% of households. The prevalence of at least one episode of diarrhea in the last 15 days was 13.6%. 34% Of children (*N* = 593) were found stunted, 11% wasted (WHZ < −2 Z-score). The proportion of children wasted increased to 13.5% when MUAC inferior to 12.5 cm was included as an additional indicator for wasting.

Demographic characteristics across the two regions were comparable, as well as presence of *G. duodenalis* infection. Parasites were found in 22.3% of the stool samples from Kratie and in 26.9% of those from Ratanak Kiri. *Giardia duodenalis* was almost the only parasite found in the stool samples (93% in Kratie and 80% in Ratanak Kiri) whereas the presence of other protozoa was found in less than 3% of the samples of both regions. No helminths were found in the stool samples from Kratie and very few in those from Ratanak Kiri (3.8%). Distribution of wealth index was approximately similar for both regions (47.8% and 53.1% poor in Kratie and Ratanak Kiri respectively and an approximately equal distribution for middle and rich). Livestock ownership differed between regions, with pig ownership more present in Ratanak Kiri (53% versus 28%, *p* < 0.001) and ownership of cows more prevalent in Kratie (62% versus 13%, *p* < 0.001). Distribution of mother`s education differed between the 2 regions with more mothers having primary (54% vs. 36%) and secondary education (35% vs. 16%), in Kratie versus Ratanak Kiri. For WASH indicators, the prevalence of improved source of drinking water and the prevalence of children who defecated in a safe place were significantly higher in Kratie compared to Ratanak Kiri (64.0% vs. 46.6% and 43.2% vs. 26.4% respectively, *p* < 0.001 for both indicators). 

The proportion of children who usually played outside the home was similar and high in both regions, with >90% of the children playing outside.

### 3.2. Associations Giardia Duodenalis Presence and Selected Covariates

Table 2, shows the results of logistic regression used to identify variables associated with the presence of *G. duodenalis*. Among the three different types of livestock ownership, pig ownership was significantly associated with *G. duodenalis* infection, with odd-ratios at 1.96 and 2.10 for un-adjusted and adjusted analysis respectively. 

**Figure 1:**
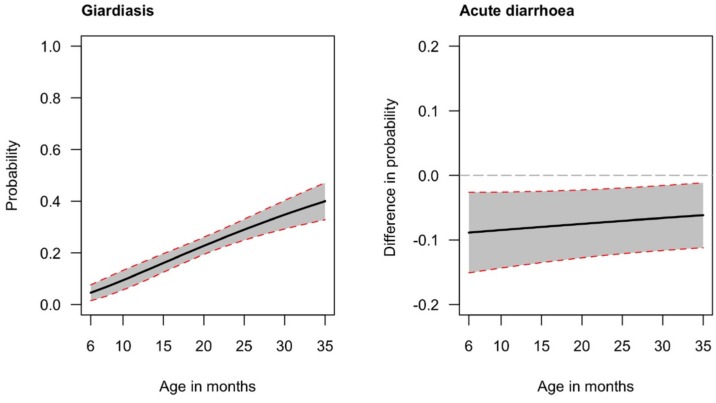
(**Left**): Predicted probability of *G. duodenalis* infection by age. (**Right**): Average marginal effect of the *giardia* variable on acute diarrhea. Both, figures were estimated using the multivariate models presented respectively in Table 2 and Table 3. Shaded areas represent 95% CI (Confidence Interval).

Wash indicators did not show strong associations. Although improved sanitation had a significant protective effect in the univariate analysis, this effect was no longer significant after adjustments (*p* values 0.021 and 0.202 respectively). Neither the place where the child usually defecated nor the source of drinking water were found associated with the presence of *G. duodenalis*. In contrast, mother’s education was associated with the presence of the protozoa. Children from a mother with secondary education were significantly less at risk of *G. duodenalis* presence than those of mothers who had no education, even after adjustment (Odd-Ratio (OR): 0.49; 95% Confident of Interval (CI): 0.26–0.92). Wealth was associated with lower presence of *G. duodenalis* in the univariate analysis (OR 0.65, *p* < 0.05), but, not in the adjusted model (*p* > 0.05). Finally, the age of the child (log transformed in the model) was strongly correlated with the presence of, with higher risk of being infected with higher age. The probability of having *G. duodenalis* infection increased from an estimated 4% at 6 months of age to 40% at 35 months of age (Figure 1, left panel).

### 3.3. Associations Acute Diarrhea Symptoms and Selected Covariates

Results of the effect of *G. duodenalis* infection on diarrhea symptoms are shown in Table 3. 

The covariates selected for the final model were: household owns pigs, wealth index, sanitation facilities, usual place where the child defecates, age and region. Surprisingly, once adjusted, the odd-ratios for *G. duodenalis* infection showed a significant protective effect on diarrhea (OR 0.45, *p* < 0.05). This significant effect is shown on the right panel of Figure 1, where the average marginal effect of the *G. duodenalis* infection on acute diarrhea is plotted for the 6 to 35 months of age period. It shows that on average the probability of a child with *G. duodenalis* infection having acute diarrhea is 7 percentage points less than for a child without the presence of *G. duodenalis* cysts. This difference is approximately constant for age, ranging from 8 percentage points at 6 months to 6 percentage points at 35 months. A significant protective effect is also observed for the wealth index variable (OR adjusted: 0.54; 95% CI: 0.32–0.92), indicating that children from richer and middle-class households are less at risk for diarrhea than those from poor families. In contrast, children living in a household that owns pigs are significantly more at risk of diarrhea even after adjustments (OR adjusted 2.00; 95% CI: 0.32–0.92). Improved sanitation facilities show a significant protective effect for diarrhea only in the unadjusted model with the association no longer significant after adjustment. 

### 3.4. Associations Anthropometric Z-Scores and Selected Covariates

Table 4 reports the results of two linear models used to study the relationship between *G. duodenalis* infection and two anthropometric outcomes: HAZ and WHZ. The final model for WHZ included as non-forced covariates: household owns pigs, mother education and sanitation facilities. The same variables were included in the final model for HAZ, in addition to mother education. The presence of *G. duodenalis* cysts was found significantly and negatively associated with HAZ only in the unadjusted model (unadjusted coeff: −0.27, *p* < 0.05).

A logistic model using the binary stunting variable (HAZ < −2) and including the same covariates was also performed and showed the same results. Indeed, *G. duodenalis* infection was a strong significant risk factors for stunting in the univariate model (OR = 1.67; 95% CI: 1.12 to 2.49), with a prevalence of stunting of 43% among children with *G. duodenalis* infection and 31% among children not infected. After inclusion of other covariates, the association between *G. duodenalis* infection and stunting was no longer significant (*p* > 0.05). Similar findings and trends were observed for pig ownership (unadjusted coeff. −0.20, *p* < 0.05). In both, the unadjusted and adjusted models, positive significant associations were noted for wealth index, mother education and sanitation facilities.

There were no significant associations between the presence of *G. duodenalis* cysts and WHZ in the unadjusted nor adjusted models. In contrast, children from households that owned pigs had a significantly lower WHZ in both, adjusted and unadjusted models (*p* < 0.001). Mother education also showed a positive effect on WHZ, with children whose mothers had secondary education or more showing a significantly higher WHZ than those with a mother with no education (*p* < 0.001). Finally, improved sanitation facilities did not show a significant effect on WHZ scores in either model.

## 4. Discussion

This study explored associations between exposure to animal feces, morbidity and anthropometric outcomes in children under 5 years of age, in Cambodia. The overall prevalence of *G. duodenalis* infection in children was found 23%. This is in line with findings from a recent study in Cambodia that presented a cumulative prevalence of 22% [15]. We believe the prevalence of 23% *G. duodenalis* infection as found in this study represents an underestimation. The 22% prevalence of *G. duodenalis* infection obtained in the recent study was calculated using different diagnostic techniques (flotation, PCR and formalin-ether concentration), while using only the formalin-ether concentration technique produced a prevalence of only 9.6% (21 out of 218 children). This study used the latter diagnostic technique. The microscopic-based technique to identify *G. duodenalis* cysts is easy and cheap, but, it lacks good levels of sensitivity. Moreover, collection of stool samples in this study was limited to one stool per child only, whereas, three consecutive stool samples in separate days are recommended for the detection of enteric parasites by microscopy due the discontinuous excretion of eggs, cysts and oocysts in time. Molecular-based techniques with high levels of sensitivity and specificity exist (for example a multiplex quantitative PCR for the presence and intensity of protozoal infection) [20], yet, their price and labor are prohibitive for most studies done in lower and middle-income countries. 

Regardless of these considerations, this study showed that household pig ownership was significantly associated with the presence of cysts of *G. duodenalis*. *Giardia duodenalis* is considered a complex species with at least eight distinct assemblages (labelled A to H), but, only assemblages A and B have been detected in both, humans and a wide range of other mammalian hosts. The other assemblages are likely to be host-specific [21]. *G. duodenalis* infection was found common in pigs, particularly in exploitations characterized by intensive farming practices [22,23,24]. In Cambodia, only one epidemiological study [15] could be found that assessed the prevalence and diversity of intestinal parasitic infections in humans and domestic animals. Surprisingly, no evidence of suine *giardiasis* was found. Transition of *G. duodenalis* between animals and humans has been reported for dogs and in a lower extent for calves and potentially pigs [25]. Further research to genotyping *G. duodenalis* would be useful to determine the most likely source of infection. 

We found a protective effect of the presence of *G. duodenalis* cysts on diarrhea, whereas *G. duodenalis* infection was also associated with lower HAZ scores in the unadjusted model. To unravel this association, it would be recommended to make molecular experiments such as Polymerase Chain Reaction (PCR) and sequencing. Unfortunately, the use of formalin for feces conservation in this study hindered the amplification of DNA. In addition, feces were recorded according to pig, cow and chicken ownership only. Further studies without formalin use and collecting information on additional animal contacts (e.g., dog feces) would be needed to elucidate the origin of the animal reservoir.

WASH indicators did not show significant associations with *G. duodenalis* infection prevalence. This may indicate that sanitation facilities, source of drinking water and usual place where the child defecates are not linked to the presence of *G. duodenalis* which is surprising for a water-borne disease. It may also indicate that indicators do not adequately reflect the risk of contamination. Low adoption of hygienic measures and consequent in-house contamination of water resources have been acknowledged as particularly risky behaviors in the literature [26].

The increase in *G. duodenalis* infection prevalence with increasing age is consistent with findings of studies carried out in preschool children in various countries [27]. The increased prevalence with age was reported to be potentially related to a child’s personal autonomy. Whereas children from 1 to 2 years of age are under surveillance most of time, those from 3 to 5 years old start to have more autonomy and are subject to less surveillance which may imply a decrease in the adoption of good hygienic practices. Consequently, older children are likely to be more exposed to risk factors and more often infected than their younger peers [27].

In regard to the association between *G. duodenalis* infection and anthropometric z-scores, positive significant associations were found for wealth index, mother education and sanitation facilities suggesting that children from a more educated mother, living in a dwelling with improved sanitation or coming from wealthy families tend to have higher HAZ. Similar factors were previously found to improve growth and nutrition status in the literature in general and in Cambodia more specifically [28,29,30]. Still, two recent large studies in Kenia and Bangladesh did not show an impact of improved WASH on anthropometry. This highlights the complexity of interactions between nutritional status, infection and hygiene [31,32].

Both, adjusted and non-adjusted odd-ratios for *G. duodenalis* infections showed a strong significant protective effect on diarrhea (Table 3). This is in line with the result of a meta-analysis of 17 studies that examined the association between diarrhea and *G. duodenalis* infection in young children in developing countries [33]. This latter analysis concluded that there was evidence of a significant adverse association between *G. duodenalis* infection and acute diarrhea. It showed that *giardiasis* was associated with a 40% lower likelihood of acute diarrhea in children from low-income countries. On the other hand, it showed that *G. duodenalis* infection is positively associated with persistent diarrhea in these populations. This is different from what is usually observed in developed countries, where children and adults are usually at risk of acute diarrhea when they encounter *G. duodenalis* infection [34,35,36]. The difference between low and middle-income countries (LMIC) vs. high income countries could be explained by the age of initial exposure and the frequency of re-exposure, as *G. duodenalis* in LMIC is endemic, leading to an initial infection in the first weeks of life and a rapid acquisition of immunity [37,38,39]. Other possible mechanisms reported to protect against symptomatic *G. duodenalis* infection in LMIC include the presence of anti- *G. duodenalis* secretory immunoglobulin A in breast-milk [40,41] and differences in the small intestine [33]. Recent observations have demonstrated that this effect may also be due to a direct immune-modulating effect of the parasite via its cathepsin B cysteine protease which cleaves pro-inflammatory chemokine, more specifically the Interleukin 8 (IL8 or chemokine (C-X-C motif) ligand 8 (CXCL8)) [42]. Robertson et al. (2010) document how new assemblages of *G. duodenalis* might cause severe symptoms when they first appears in a population. The latter could contribute to explaining the contradictory literature on the relationship between diarrhea and *G. duodenalis* infection [43]. The immune status of the host and the “strain” of the parasite appear to influence susceptibility to infection, as well as the severity of clinical signs [43]. In addition, *G. duodenalis* infection may increase the virulence of commensal microbiota bacteria during the acute phase of the infection. In turn, these altered microbiota induce a host inflammatory response, which may be responsible, at least in part, for the long-term, post-infectious, complications seen in *giardiasis* [42]. *Giardia duodenalis* infection could protect against diarrhea, for example by competing with or suppressing other enteric pathogens (virus, bacteria or parasite), or by inducing changes in mucosal immunity [43].

In this study, a significant univariate association was noted for the presence of *G. duodenalis* and HAZ scores, but, this association was not seen after adjustments. The positive association between stunting and *G. duodenalis* infection is in line with several studies that have shown that *G. duodenalis* infection may impair linear growth, presumably by reducing intake and/or causing malabsorption of nutrients [10,43,44]. Children with *G. duodenalis* infection were found to have lower serum zinc and iron status, both acknowledged for their importance in growth [45,46]. The association between livestock and WHZ was found significant, yet, not for HAZ. This is in line with results in the literature that report presence of livestock and improved HAZ because the livestock is a source of food and nutrition, yet, it may also represent a risk from exposure to animal feces harmful to child nutrition status [26].

Our analysis showed that pig ownership represents an increased risk of having acute diarrhea (Table 3). Significant positive associations between livestock and diarrhea illness among children and adults have been documented in the literature. A systematic review of 29 studies examining the association between domestic animal husbandry and diarrheal infections found consistent evidence of a positive association [8]. On the other hand, a recent assessment of Demographic Health Surveys (DHS) from 30 sub-Saharan African countries found an inconsistent relationship between childhood diarrhea and household livestock [47]. Among the countries included, 14 indicated livestock ownership as a risk factor whereas 10 exhibited a protective association. 

The present study builds on data from the My Health project, a longitudinal study that was not specifically designed for the current research and data should be considered observational in nature. We acknowledge that this study may be subject to bias. Including a selection bias from mothers who did not submit stool samples of their children reportedly because they did not defecate during the proposed timespan (approximately 24 h). Also, recall bias may have occurred where mothers where asked to report on acute diarrhea episodes in their children over the last two weeks. Finally, although models were adjusted for a series of confounders, it is possible that results are biased by unobserved covariates such as factors related to food consumption.

## 5. Conclusions

The adverse outcomes of malnutrition on the development of a child are well acknowledged as are the broad variety of contextual factors that may impact on child nutritional status. Improved sanitation and protection from human feces can significantly lower the incidence of diarrhea and environmental enteropathy. However, the impact of excessive exposure to animal feces on child’s health and development is less well documented.

We showed that the presence of livestock and more particularly pigs near the main household dwelling is a risk factor for *G. duodenalis* infection. Although, presence of *G. duodenalis* was found a protective factor for acute diarrhea, it was associated with stunting. This finding may be indicative of a subclinical condition like EED. The diagnosis and treatment of *G. duodenalis infection* is complex in LMIC, such as Cambodia (affordability, feasibility, acceptability) and preventive measures that avoid extensive exposure of young children to animal feces may considerably protect against stunting and improve potential for a healthy development. Programs that focus on animal containment, proper animal waste management, strengthened veterinary control of animal health as well as improved adoption of good hygiene practices, and more particularly frequent hand washing with soap could be most effective in a context similar to Cambodia. 

The findings also highlight a need for revised metrics and indicators that do not sufficiently consider the dynamics of environmental transmission of pathogens within households [48]. The results support the integration of cross-sector policy measures that reinforce comprehensive early childhood interventions towards improving nutritional status as part of a wider set of child welfare and development measures.

## Figures and Tables

**Table 1:** Summary of the selected sample characteristics.

	Kratie	Ratanak Kiri	Overall	*p* Value ^†^
Full sample				
*N*	378	322	700	
Child play outside, % (SE)	91.8 (1.4)	90.7 (1.6)	91.3 (1.1)	0.594
Study sample				
*N*	347	292	639	
Demographic variable				
Age in months, mean (SD)	20.7 (±8.2)	20.6 (±8.1)	20.6 (±8.2)	0.854
Boys, % (SE)	50.1 (2.7)	49.0 (2.9)	49.6 (1.2)	0.768
Morbidity				
Giardia Duodenalis, % (SE)	23.6 (2.3)	22.3 (2.4)	23 (1.7)	0.682
Had diarrhea last 15 days, % (SE)	7.2 (1.4)	21.2 (2.4)	13.6 (1.4)	0.000
Animal owned				
Pig, % (SE)	27.7 (2.4)	52.7 (2.9)	39.1 (1.9)	0.000
Cow, % (SE)	62.2 (2.6)	13.4 (2)	39.9 (1.9)	0.000
Chicken, % (SE)	10.4 (1.6)	18.2 (2.3)	13.9 (1.4)	0.005
Socio-economic variables				
Wealth index, % (SE)				0.414
Poor	47.8 (2.7)	53.1 (2.9)	50.2 (2)	
Middle	28.5 (2.4)	26 (2.6)	27.4 (1.8)	
Rich	23.6 (2.3)	20.9 (2.4)	22.4 (1.7)	
Mother education, % (SE)				0.000
No education	10.7 (1.7)	47.9 (2.9)	27.7 (1.8)	
Primary	53.6 (2.7)	36 (2.8)	45.5 (2.0)	
Secondary +	35.7 (2.6)	16.1 (2.2)	26.8 (1.8)	
Wash variables				
Improved sanitation facilities, % (SE)	36.0 (2.6)	31.2 (2.7)	33.8 (1.9)	0.196
Improved source of drinking water, % (SE)	64.0 (2.6)	46.6 (2.9)	56.0 (2.0)	0.000
Child usually defecates in a safe place, % (SE)	43.2 (2.7)	26.4 (2.6)	35.5 (1.9)	0.000
Anthropometric sample				
*N*	302	291	593	
HAZ, mean (SD)	−1.38 (±1.04)	−1.74 (±1.06)	−1.56 (±1.06)	<0.001
Stunting, % (SE)	26.5 (2.5)	41.9 (2.9)	34.1 (1.90)	<0.001
WHZ, mean (SD)	−0.81 (±0.95)	−1.08 (±0.94)	−1.05 (±0.95)	<0.001
MUAC, mean (SD)	14.07 (±1.05)	13.74 (±1.11)	13.91 (±1.09)	<0.001
Wasting: WHZ < −2, % (SE)	9.9 (1.7)	12.7 (2.0)	11.3 (1.3)	0.285
Wasting ^‡^: WHZ < −2 and/or MUAC < 12.5, % (SE)	11.3 (1.8)	15.8 (2.1)	13.5 (1.4)	0.105

SD = Standard Deviation. SE = Standard Error. HAZ = Height for Age Z-scores. WHZ = Weight for Height Z-scores; MUA = Middle Upper Arm Circonference; ^†^ Testing differences in the distribution of the variable between the two regions: Chi-square test for categorical variable and one-way anova for normally distributed variables; ^‡^ Following WHO definition, the MUAC cut-off was applied for children who are more than 6 months.

**Table 2:** A logistic regression model explaining the presence of *G. duodenalis* in children.

	*G. duodenalis*							
	*N*	% ^c^	Unadjusted ^a^			Adjusted ^b^		
			Odd-Ratio	C.I.	*p* Value	Odd-Ratio	C.I.	*p* Value
Animals owned								
Pig								
No	389	18.2	1.00	-		1.00	-	
Yes	250	30.4	1.96	(1.35, 2.84)	<0.001	2.10	(1.33, 3.30)	0.001
Cow								
No	384	21.1	1.00	-		1.00	-	
Yes	255	25.9	1.31	(0.90, 1.90)	0.16	1.15	(0.71, 1.85)	0.577
Chicken								
No	89	20.2	0.83	(0.48, 1.44)	0.502	1.08	(0.59, 2.00)	0.800
Yes	550	23.4	1.00	-		1.00	-	
Socio-economic position								
Wealth index ^d^								
Poor	321	26.8	1.00	-		1.00	-	
Middle/Rich	318	19.2	0.65	(0.45, 0.94)	0.023	0.86	(0.56, 1.30)	0.467
Mother-education								
No education	177	28.2	1.00	-		1.00	-	
Primary	291	24.4	0.82	(0.54, 1.25)	0.357	0.91	(0.55, 1.51)	0.725
Secondary	171	15.2	0.46	(0.27, 0.77)	0.004	0.49	(0.26, 0.92)	0.027
Wash variable								
Sanitation facilities								
Non improved	423	25.8	1.00	-		1.00	-	
Improved	216	17.6	0.61	(0.41, 0.93)	0.021	0.73	(0.45, 1.19)	0.202
Source of drinking water								
Non improved	281	25.6	1.00	-		1.00	-	
Improved	358	20.9	0.77	(0.53, 1.11)	0.164	1.1	(0.72, 1.70)	0.657
Usual place child defecates								
Unsafe	412	25.2	1.00	-		1.00	-	
Safe	227	18.9	0.69	(0.46, 1.03)	0.071	1.08	(0.66, 1.76)	0.759
Demographic variable								
Age in months ^e^	639	-	4.13	(2.49–6.87)	<0.001	4.72	(2.77, 8.05)	<0.001
Region								
Kratie	347	23.6	1.00	-		1.00	-	
Ratanak Kiri	292	22.2	0.93	(0.64, 1.34)	0.682	0.7	(0.42, 1.19)	0.186

^a^ Unadjusted: univariate analysis; ^b^ Adjusted for all the covariates included in the model; ^c^ Prevalence of *G. Duodenalis*; ^d^ Middle and Rich categories were merged together because their associated odd-ratio were similar; ^e^ Log transformed variable.

**Table 3:** Logistic regression model for the association between acute diarrhea and selected covariates.

	Diarrhea							
	*N*	%^c^	Unadjusted ^a^			Adjusted ^b^		
			Odd-Ratio	C.I.	*p* Value	Odd-Ratio	C.I.	*p* Value
Giardia								
No	492	15.0	1.00	-		1.00	-	
Yes	147	8.8	0.55	(0.29, 1.02)	0.058	0.45	(0.23, 0.87)	0.017
Have Pig								
No	385	8.5	1.00	-		1.00	-	
Yes	254	21.6	2.34	(1.45, 3.80)	0.001	2.00	(1.19, 3.35)	0.009
Wealth index ^d^								
Poor	321	18.4	1.00	-		1.00	-	
Middle/Rich	318	8.8	0.43	(0.27, 0.69)	0.001	0.54	(0.32, 0.92)	0.023
Sanitation facilities								
Non improved	423	16.3	1.00	-		1.00	-	
Improved	216	8.3	0.48	(0.28, 0.84)	0.011	0.71	(0.38, 1.33)	0.286
Usual place child defecates								
Unsafe	412	17.0	1.00	-		1.00	-	
Safe	227	7.5	0.48	(0.27, 0.84)	0.010	0.73	(0.38, 1.39)	0.34
Age in months	639	-	0.97	(0.95, 1.00)	0.056	0.98	(0.95, 1.01)	0.211
Region								
Kratie	347	7.2	1.00	-		1.00	-	
Ratanak Kiri	292	21.2	3.42	(2.12, 5.69)	<0.001	2.69	(1.59, 4.55)	<0.001

^a^ Have Pig, sanitation facilities and usual place child defecates were adjusted for region; ^b^ Adjusted for all the covariates in the model; ^c^ Prevalence of diarrhea; ^d^ Middle and rich categories were merged together because their associated odd-ratios were similar.

**Table 4:** Linear regression models explaining the associations between anthropometric z-score and selected covariates.

	HAZ ^†^							WHZ ^‡^					
	*N*	Unadjusted ^a^			Adjusted ^b^			Unadjusted ^a^			Adjusted ^b^		
		Coeff.	C.I	*p* Value	Coeff.	C.I.	*p* Value	Coeff.	C.I	*p* Value	Coeff	C.I.	*p* Value
Giardia													
No	464	-	-	-	-	-	-	-	-	-	-	-	-
Yes	129	−0.27	(−0.47, −0.06)	0.012	−0.07	(−0.27, 0.13)	0.501	−0.06	(−0.25, 0.12)	0.516	0.10	(−0.09, 0.28)	0.317
Have Pig													
No	364	-	-	-	-	-	-	-	-	-	-	-	-
Yes	229	−0.20	(−0.38, −0.01)	0.035	−0.05	(−0.23, 0.13)	0.595	−0.23	(−0.40, −0.07)	0.005	−0.21	(−0.37, −0.04)	0.014
Wealth index													
Poor	290	-	-	-	-	-	-						
Middle	167	0.30	(0.10, 0.50)	0.003	0.18	(−0.02, 0.38)	0.071						
Rich	136	0.56	(0.34, 0.77)	<0.001	0.38	(0.16, 0.6)	<0.001						
Mother Education													
No education	171	-	-	-	-	-	-	-	-	-	-	-	-
Primary	263	0.31	(0.09, 0.52)	0.005	0.20	(−0.01, 0.41)	0.068	0.11	(−0.09, 0.30)	0.278	0.05	(−0.14, 0.24)	0.600
Secondary and more	159	0.56	(0.32, 0.81)	<0.001	0.41	(0.16, 0.66)	<0.001	0.30	(0.08, 0.52)	0.008	0.27	(0.05, 0.50)	0.019
Sanitation facilities													
Non improved	389	-	-	-	-	-	-	-	-	-	-	-	-
Improved	204	0.36	(0.18, 0.53)	<0.001	0.20	(0.02, 0.38)	0.032	0.15	(−0.01, 0.31)	0.059	0.10	(−0.06, 0.26)	0.226
Gender													
Female	301	-	-	-	-	-	-	-	-	-	-	-	-
Male	292	−0.13	(−0.31, 0.04)	0.126	−0.15	(−0.31, 0.01)	0.067	0.00	(−0.15, 0.15)	0.995	0.01	(−0.14, 0.16)	0.912
Region													
Female	302	-	-	-	-	-	-	-	-	-	-	-	-
Male	291	-0.35	(−0.52, −0.18)	<0.001	−0.18	(−0.36, 0.00)	0.050	−0.28	(−0.43, −0.12)	<0.001	−0.14	(−0.31, 0.02)	0.093

HAZ = Height for Age z-scores. WHZ= Weight for Age z-scores. Wealth index variables was not included in the final model for WHZ; ^†^ R^2^ for adjusted HAZ model: 14.2 %, *p*-Value of the model: 0.00001; ^‡^ R^2^ for adjusted WHZ model: 9.1%, *p*-Value of the model: 0.00001; ^a^ Have Pig, mother education and sanitation facilites were adjusted for region; ^b^ Adjusted for all the covariates in the model, Age and Age squared (coefficients not shown).
